# Interaction between reflected shock waves and laser-induced cavitation bubbles

**DOI:** 10.1016/j.ultsonch.2026.107842

**Published:** 2026-04-01

**Authors:** Mazyar Dawoodian, Sasan Rezaee, Dipanjan Barman, Ould el Moctar, Rafael Manso Sainz, Robert Mettin, Christiane Lechner

**Affiliations:** a Institute for Sustainable and Autonomous Maritime Systems, University of Duisburg-Essen, 47057 Duisburg, Germany; b Drittes Physikalisches Institut, Universität, Göttingen, Friedrich-Hund-Platz 1, 37077 Göttingen, Germany; c Institute of Fluid Mechanics and Heat Transfer, TU Wien, Getreidemarkt 9, 1060 Vienna, Austria; d Autonomous and Energy-efficient Maritime Technologies, Technische Universität Berlin, Müller-Breslau-Str. 15, 10623 Berlin, Germany

**Keywords:** Cavitation, Laser-induced bubble, Shock-wave echoes

## Abstract

This study experimentally investigates the interaction between acoustically reflected shock-wave echoes and laser-induced cavitation bubbles confined within cylindrical tubes. In these macroscopic experiments, the tube radius was systematically varied to control the timing and amplitude of reflected shocks relative to the bubble lifetime. Time-resolved high-speed imaging captured the bubble’s expansion and collapse dynamics, revealing that early reflected echoes, returning within microseconds after the initial breakdown, strongly influence the collapse symmetry and morphology. Depending on their timing and intensity, these echoes compress the bubble along the tube axis, leading to prolate deformations and extended collapse durations. To complement these findings at the nanoscale, molecular dynamics (MD) simulations were performed to examine the interaction between plasma-induced shock waves and nanobubbles within cylindrical confinement. The simulations show that higher-energy pulses generate strong rebound shocks that induce prolate deformation and secondary growth–collapse cycles. Together, the experimental and atomistic results reveal a consistent physical mechanism across scales, where confinement-induced shock reflections modulated bubble dynamics through the timing and amplitude of early reflected waves. These findings provide new insight into echo-driven cavitation in confined geometries, with potential implications for ultrasonic cleaning, biomedical cavitation, and focused acoustic applications.

## Highlights

• Acoustically reflected shock-wave echoes in confined geometries strongly modulate laser induced cavitation bubble dynamics.
• Experiments reveal echo-driven prolate deformation.
• Molecular dynamics simulations confirmed echo-driven impact on nanobubble dynamics.

## Graphical abstract

Time-resolved visualization of a laser-induced cavitation bubble in a cylindrical tube, showing how successive acoustically reflected shock-wave echoes progressively compress the bubble and induce a prolate, axially focused collapse.

**Figure:**
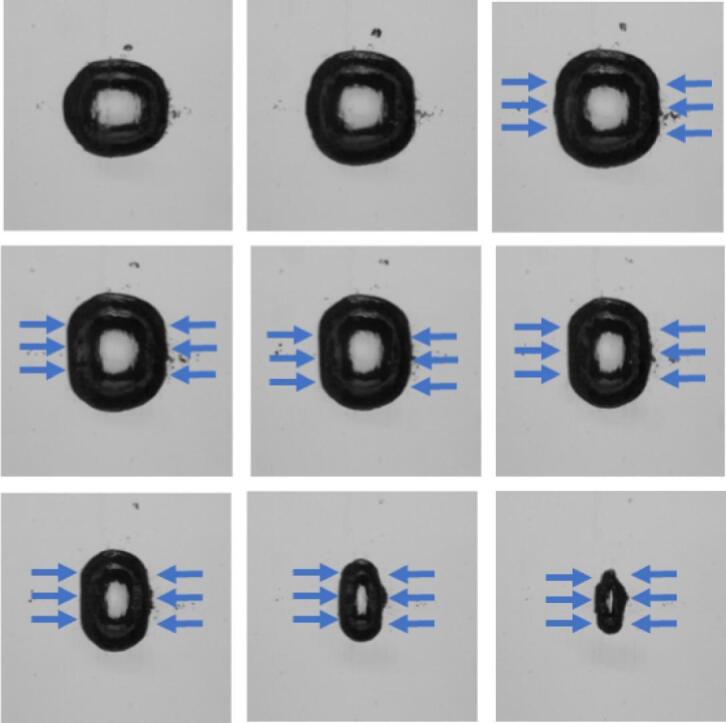

## 1 Introduction

The interaction of bubbles with pressure waves is a complex multiphase phenomenon occurring in a wide variety of fluid systems, ranging from turbomachinery and hydrodynamic devices to biomedical and acoustic applications[1], [2], [3], [4], [5], [6], [7]. When a pressure wave impinges on a bubble, the sudden pressure discontinuity accelerates the liquid–gas interface, leading to rapid compression or collapse and, under certain conditions, the formation of high-speed liquid jets[8], [9], [10], [11], [12], [13], [14], [15]. The direction and strength of these jets are governed by the asymmetry of the surrounding flow and by nearby boundaries: the bubble typically jets toward a rigid surface, splits near an elastic surface, or jets away from a free surface[4]. In case of shock-bubble interaction, the acceleration imposed by the passing shock may reach several km/s, producing highly asymmetric collapses accompanied by supersonic microjets and intense localized pressure pulses[16], [17], [18]. Such violent dynamics generate secondary shock waves and strong stress concentrations that can either be exploited or must be mitigated, depending on their application. In biomedical and industrial contexts, these phenomena underpin ultrasonic cleaning, lithotripsy, high-intensity focused ultrasound (HIFU) therapy, and targeted drug delivery[19], [20], [21], [22], while in engineering systems they contribute to cavitation erosion, pressure surges, and flow-induced vibration[23], [24].

Experiments by Sankin et al. (2005)[17] demonstrated that the phase of bubble oscillation at the moment of shock arrival plays a decisive role in the collapse intensity. When a lithotripter shock reaches a laser-generated bubble during its collapse phase, the resulting compression is up to five times stronger than when the same shock arrives during expansion, producing pronounced non-spherical deformation and a fast axial jet. Their findings confirmed that bubble dynamics under transient shock loading depend not merely on the pressure amplitude, but on the timing of the impulsive acceleration relative to the bubble’s natural oscillation. Subsequent studies further revealed that nearby boundaries and elastic substrates modify the pressure field and enhance jet penetration or redirect collapse asymmetry[25]. Complementary insights were provided by Ohl et al. (2015)[4], who reviewed single- and multi-bubble interactions with shock and ultrasound waves. They highlighted that even in nominally symmetric acoustic fields, bubbles undergo strongly nonlinear oscillations that frequently culminate in jet formation and secondary shock emission. The jet direction is dictated by the resistance of adjacent materials, while its velocity, typically hundreds to thousands of m s⁻^1^, is largely independent of bubble size. In high-intensity focused ultrasound, dense bubble clouds exhibit collective behaviour governed by radiation (Bjerknes) forces[26], [27], leading to shielding, synchronization, and complex “comet-like’’ or “umbrella’’ structures. These observations demonstrated that bubble wave coupling is highly sensitive to confinement and repeated acoustic forcing, as in tubes or biological tissues, where reflections amplify subsequent interactions.

In the classical free-field configuration[28], a single impulsive shock interacts once with an initially quiescent bubble. However, in confined geometries, such as tubes, the shock wave reflects at the boundaries and refocuses toward the center, creating a sequence of secondary echoes that may re-impact the same bubble within microseconds of the initial event. These acoustically reflected and refocused waves constitute a distinct driving mechanism, capable of periodically modulating the bubble’s oscillation through constructive or destructive interference with its natural dynamics. The resulting interactions between successive shock echoes and the bubble lead to complex behaviors, including asymmetric collapses, delayed rebounds, and complex surface deformations, which are not captured by standard Rayleigh–Plesset or Keller–Miksis formulations[29], [30], [31].

Recent high-resolution studies of shock-bubble dynamics provided new physical insight into such transient processes. Experiments and simulations revealed that coupling between acceleration-driven interfacial instabilities and bubble collapse governs the onset of jet formation and bubble deformation[9], [10], [11], [12], [32]. The Richtmyer-Meshkov and Rayleigh-Taylor instabilities, in particular, describe how impulsive or sustained accelerations of the liquid–gas interface may generate jets and spikes similar to those observed in shock-driven bubble collapses[33], [34], [35], [36], [37]. Within this context, the confined reflections in a tube act as temporally separated acceleration impulses, repeatedly driving the interface and potentially amplifying or suppressing the deformation of the bubble, depending on their phase relative to the bubble oscillation.

The study of echo-driven shock-bubble interactions, therefore, extends classical cavitation theory into a regime where multiple, geometry-dependent pressure waves interact with a single bubble. Understanding these interactions is not only of fundamental importance for cavitation dynamics, but also for optimizing technologies that rely on confined or focused acoustic fields, such as ultrasonic cleaning systems, biomedical sonication, and laser-based microfluidic actuation. Despite their relevance, quantitative data on how echo timing and amplitude affect the evolution of laser-induced cavitation bubbles remain scarce, primarily because of the experimental difficulty of resolving rapid successive shocks within a confined domain.

In the case of laser-induced cavitation, the initial optical breakdown generates a strong, spherically diverging shock wave and a rapidly expanding plasma bubble[1], [38]. As the shock propagates within a confined geometry, such as a cylindrical tube, part of its energy is reflected at the walls and subsequently refocused toward the tube center. These reflections give rise to secondary pressure pulses or echoes, which arrive at discrete times determined by the tube radius and the speed of sound in the liquid. Each echo imposes a transient pressure disturbance on the bubble that remains from the primary event. Depending on the relative phase between the echo arrival and the bubble’s oscillatory motion, these secondary shocks either reinforce or oppose the collapse, causing stronger compression, delaying the rebound and promoting asymmetric deformations.

While single-bubble dynamics under laser confinement have been studied in various geometries, including near rigid or free surfaces[8], [39], [40], [41], the specific influence of repeated, geometry-controlled shock echoes on bubble behavior has not been systematically explored. The presence of multiple echoes introduces an additional level of temporal control over the driving pressure field, offering a unique opportunity to modulate the bubble collapse process without altering the initial laser energy. This mechanism is particularly relevant in microfluidic and biomedical contexts, where reflective boundaries or tissue interfaces naturally generate secondary pressure waves that can interact with cavitation nuclei on microsecond time scales[21], [42], [43].

Beyond classical shock–bubble interaction in unbounded or near-wall configurations, recent studies have emphasized the significant role of geometric confinement in modifying cavitation dynamics. Analytical and numerical works have shown that cylindrical confinement modifies the inertial response of a bubble even when it remains nearly spherical. Zudin[44] derived a cylindrical analog of the Rayleigh equation demonstrating additional liquid-column inertia in tubes, while Oguz and Prosperetti[45] showed that the natural oscillation frequency depends explicitly on tube radius. Different modification of the Rayleigh model were further analysed by Klotz and Hyninen[46]. Yuan et al.[47] further reported that bubble lifetime and maximum volume in small channels are strongly affected by tube aspect ratio. Experimental studies, primarily conducted in very narrow tubes or thin-gap geometries, have confirmed that strong confinement markedly alters bubble lifetime, deformation, and jet formation. Wang et al.[48] demonstrated that bubbles inside rigid circular tubes exhibit pronounced axial elongation and modified jet development under confinement. Wang et al.[49] demonstrated that wall confinement in thin tubes strongly affects bubble lifetime, deformation, and jet formation, with tube diameter emerging as a critical control parameter. Li et al.[50] experimentally investigated spark-induced cavitation bubbles in rigid tubes and showed that tube diameter and boundary proximity markedly influence collapse morphology and rebound behavior. In confined planar geometries, Gebensleben et al.[51] reported substantial changes in collapse timing and flow structures due to geometric restriction. Similarly, Wang et al.[52] observed that narrow-gap confinement significantly alters bubble deformation and jet development compared with less confined conditions. These findings underline that confinement geometry plays a central role in cavitation bubble evolution. However, while previous studies primarily examined wall proximity or gap confinement effects, the influence of tube diameter on repeated shock-wave reflections and echo-driven bubble modulation has not been systematically addressed. The present study therefore focuses on pipe diameter as a governing parameter controlling echo timing, impulsive forcing, and collapse anisotropy.

In this study, we experimentally investigated the dynamics of laser-induced cavitation bubbles confined within cylindrical tubes, focusing on how acoustically reflected and refocused shock-wave echoes influence bubble evolution. Our investigation emphasized the role of secondary echoes arising from reflections at the tube wall and their effect on bubble dynamics. By varying the tube radius, the timing and amplitude of reflected shocks relative to the bubble lifetime were controlled. High-speed imaging at sub-microsecond resolution was employed to capture the bubble’s growth and collapse phases and to identify deformation modes associated with successive echo interactions. The results elucidated how echo timing and confinement geometry govern the symmetry and morphology of the collapsing bubble, revealing the mechanisms responsible for the observed prolate collapse shape. Our findings provided new physical insight into echo-driven modulation of cavitation dynamics and, hopefully, contribute to improved designs of advanced acoustic and laser systems, where controlled cavitation plays a functional role, including ultrasonic cleaning and high-intensity focused acoustic applications.

Furthermore, in case of nanobubbles (nanoscale bubbles), their intensive properties and qualitative behavior are comparable to those of micro- and millimeter-sized bubbles. However, their extensive properties, such as size, lifetime, and response to external forces like shock waves, exhibit distinct and unique phenomena due to the high surface-to-volume ratio at nanoscale[53]. Additionally, nanobubbles generally exhibit higher population densities and longer lifetimes, lasting several weeks, thereby underscoring their importance in the study of cavitation under shock wave conditions[54], [55], [56]. Accordingly, one of the objectives of our present study was to investigate the propagation of shock waves (specifically, rebound shocks generated by plasma near rigid walls) and their effects on nanobubble dynamics. To achieve this, molecular dynamics (MD) simulations were performed. The results we obtained provided insight into the distinct effects of shock waves on nanobubbles compared to millimeter-scale bubbles and visualized the dynamic response of nanobubbles to shock wave interaction.

## 2 Experimental setup

**Fig. 1:**
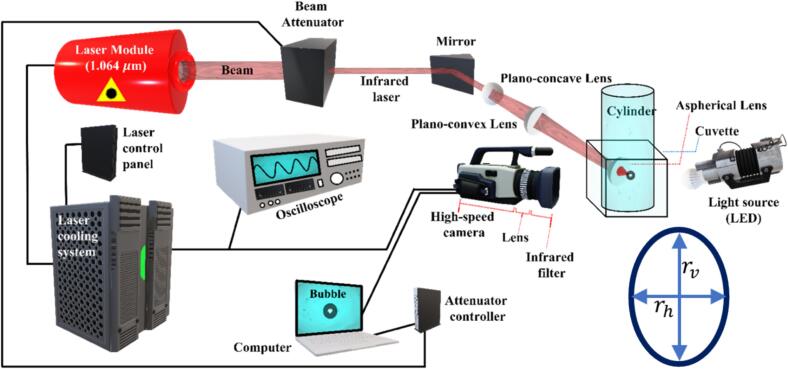
Schematic diagram of the experimental setup, illustrating the generation of a cavitation bubble and the imaging using shadowgraphy. The guided infrared laser focused at the center of the cuvette using a sequence of mirror and lenses. The focused infrared laser generated high-energy plasma that created the cavitation bubble. The bubble was illuminated with a diffused LED light (perpendicularly to the path of infrared laser), and the shadowgraph images were captured with a synchronised high-speed camera. The lower-right inset depicts a schematic representation of the bubble, where $r_{h}$ and $r_{v}$ denote the instantaneous horizontal and vertical radii used to quantify bubble deformation and aspect ratio ($r_{v}/r_{h}$) during oscillation.

The experiments were carried out in the laser laboratory of the Institute for Sustainable and Autonomous Maritime Systems at the University of Duisburg-Essen. A single cavitation bubble was generated in distilled water inside an acrylic cuvette by focusing a high-energy pulsed Nd:YAG infrared laser beam. The optical setup, test chamber, and high-speed imaging system are described below, and a schematic diagram of the experimental setup is shown in Fig. 1. The cavitation bubble was generated using a Q-Smart 450 Nd:YAG infrared laser (Quantel, France), operating at a wavelength of 1064 nm with a pulse duration of 6 ns and a maximum pulse energy of 450 mJ. The laser was operated in single-shot mode. A motorized attenuator was used to regulate the transmitted pulse energy between 5% and 100% of the maximum available energy (450 mJ), which also represents the highest pulse energy used in the experiments, allowing reproducible generation of bubbles of various sizes. After attenuation, the laser beam was redirected by a 90 deg mirror and passed through a pair of collimating lenses before being focused into the water volume by an aspheric lens (f = 25 mm). The focal point was located at the center of the cuvette, resulting in a localized optical breakdown and subsequent nucleation of a cavitation bubble. The test chamber consisted of a transparent acrylic cuvette with internal dimensions of 50 × 50 × 50 mm^3^ and wall thickness 10 mm. The laser beam entered through one wall and was focused at mid-depth along the central vertical axis. No external flow or mechanical agitation was applied, ensuring a quiescent liquid environment for the bubble dynamics. Inside the cuvette, a cylindrical acrylic insert with various radii between 8 and 18 mm was placed concentrically. The laser beam was focused such that cavitation bubbles were generated on the cylinder axis. Water was filled both inside the acrylic cylinder and in the annular region between the cylinder and the cuvette walls. The water was maintained at ambient laboratory conditions, with a temperature of about $20^{{}^{\circ}}C$ and an atmospheric pressure of 101 kPa.

Bubble dynamics were captured using a high-speed camera (Phantom v2012) positioned perpendicular to the laser axis. A high-energy diffused LED (60 W) backlight provided shadowgraph illumination. The resolution was 256×256 pixels, and the frame rate was 180,000 fps with an exposure time of 0.86 µs, which was sufficient to resolve the fast expansion and collapse phases. The laser and the camera were synchronized via a TTL trigger pulse from the laser controller to ensure precise timing. To ensure repeatability, multiple experiments were conducted for each tube radius and pulse energy setting. The maximum equivalent bubble radius was reproducible within ± 0.1 mm, and the observed deformation and collapse trends were consistent across repeated runs. Adequate time was allowed between shots to eliminate residual flow effects, ensuring stable experimental conditions.

## 3 Results and discussion

### 3.1 Shock-wave propagation and echo formation

Immediately after optical breakdown, the laser focus region transformed into a dense plasma, producing a localized zone of extremely high temperature and pressure. The near-source overpressure was characterized by pressure $p_{0}$ at an effective plasma radius $r_{0}$. For nanosecond laser pulses in water, $r_{0}$ was typically of the order of 30 to 80 μm, depending on focusing conditions and pulse energy[57], [58], [59]. The initial shock pressure near the plasma boundary reached 0.5 to 5 GPa, with a representative value of $p_{0}\approx 1GPa$ for moderate pulse energies[57], [58], [59]. This pressure decayed rapidly with distance due to geometric spreading and viscous-thermal attenuation, and then it reduced to megapascal levels within a few hundred micrometres from the focus[57], [58], [59], [60]. These parameters $p_{0}$ and $r_{0}$ served as the initial conditions to model the outward-propagating shock wave and its subsequent reflections within the tube. The propagation of the pressure wave in water was described as a spherically spreading pulse subject to weak thermoviscous attenuation. Following the classical acoustic formulation for spherical waves in an absorbing medium[61], the pressure amplitude decays with distance according to

(1) $$p\left(r\right)=p_{0}\frac{r_{0}}{r}\;e^{-\alpha_{eff}\left(r-r_{0}\right)},$$

where α is the effective attenuation coefficient of water. The 1/r term arises from conservation of acoustic energy over a spherical wavefront, while the exponential term accounts for viscous and thermal losses. This model is consistent with the classical analysis by Pierce[61], who derived similar spatial dependence for spherical spreading in lossy media.

Upon reaching the inner wall of the cylindrical tube of radius R, the shock wave was partially reflected due to the acoustic impedance mismatch between water and Plexiglas (PMMA). Upon reflection, the wave undergoes geometric refocusing toward the tube axis as a consequence of cylindrical confinement. In the plane of the tube cross section, the reflected wavefronts converge toward the center rather than propagating isotropically as in a free field. As the reflected wave travels inward, the effective wavefront area decreases, leading to an amplification of the local pressure amplitude near the tube axis and producing a train of successive echoes. The combined effects of geometric spreading, exponential attenuation, impedance reflection, and geometric focusing determined the pressure amplitude of the k-th echo at the bubble center, expressed as

(2) $$p_{c}^{\left(k\right)}=G\;p_{0}\frac{r_{0}}{R}\;e^{-{2\alpha }_{eff}R}\;\beta \Gamma \;{\left[\beta \Gamma \;\;e^{-2\alpha_{eff}R}\right]}^{k-1},\left(k=1,2,\cdots \right).$$

G is the geometric focusing gain, representing the pressure amplification associated with the convergence of the reflected wavefronts. This gain can be interpreted as the ratio between the characteristic radial extent of the tube and the effective focal width (Δ) over which the reflected pressure pulse is concentrated (G=R/Δ). Physically, this focal width represents the finite spatial region around the tube axis where the refocused shock energy is distributed, rather than an idealized point focus.

**Fig. 2:**
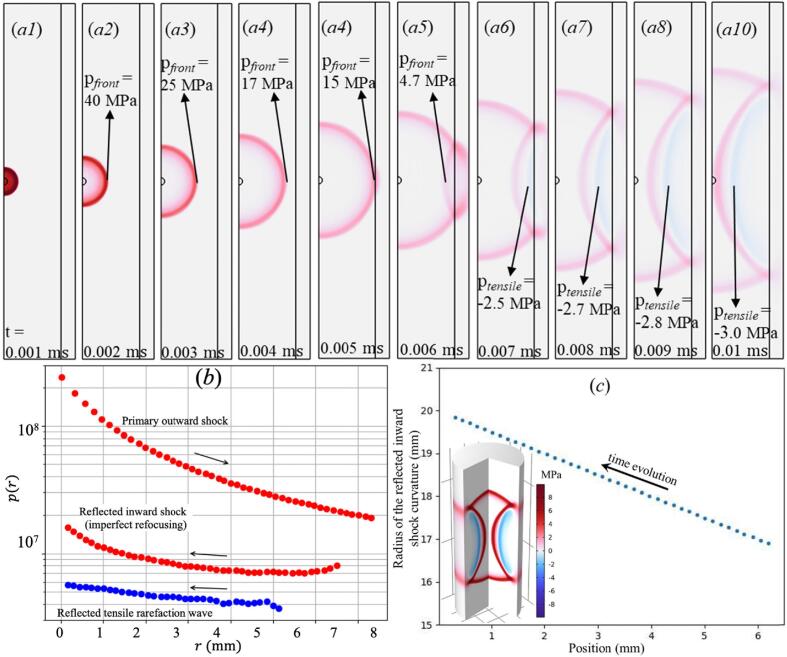
(a) Simulated propagation of an acoustic shock wave in a cylindrical cuvette (R=8 mm) generated by a monopolar mass source activated at *t* = 0. Screenshots are shown at successive times, illustrating outward propagation, partial reflection at the PMMA wall, and refocusing of the reflected wave toward the center. (b) Radial distribution of the 1st echo pressure amplitude $p_{c}^{\left(k\right)}/p_{0}$ for tube size 8 mm (the arrows indicate time evolution). The upper (red) markers show the outward-propagating primary shock wave, the middle (red) markers show the generated inward-propagating shock, and the lower (blue) points represent the reflected tensile (rarefaction) wave. (c) Time evolution of the radius of curvature of the focusing shock wave near the tube axis (R = 8 mm). The curvature radius remains larger than the bubble radius, indicating that the incoming shock can be considered cylindrically symmetric to a good approximation. The inset shows a 3D visualization of the shock structure at t=9.5μs, shortly before reaching the focus on the axis, illustrating the confined cylindrical geometry of the focusing shock.

In practice, the refocusing in the tube is not perfectly spherical: while the wave converges in the radial plane, it continues to diverge in the axial direction. To account for this imperfect refocusing, a correction factor β is introduced, representing the fraction of the geometric focusing gain that is effectively realized. Based on the COMSOL simulations of the pressure field (see below), this factor was estimated as β≈0.135, relatively independent of the cylinder radius. The resulting pressure amplification therefore scales with Γβ. The geometric focusing gain thus captures the pressure enhancement due to cylindrical wave convergence in the cross-sectional plane, while the factor β accounts for the additional loss associated with incomplete three-dimensional focusing. Γ is the reflection coefficient at the water-Plexiglas interface, and $\alpha_{eff}$ is the effective attenuation coefficient. Here, $p_{0}=1GPa$ and $r_{0}=50\mu m$ are representative plasma parameters. The amplitude reduction due to wall reflections was evaluated using the pressure reflection coefficient

(3) $$\Gamma =\frac{Z_{PMMA}-Z_{water}}{Z_{PMMA}+Z_{water}},$$

where Z=ρc is the acoustic impedance, and ρ and c are, respectively, the density and speed of sound in the unperturbed medium. With $Z_{water}\approx 1.48\;\text{MRayl}$ and $Z_{PMMA}\approx 3.2\;\text{MRayl}$, the resulting Γ≈0.37. The effective attenuation coefficient α can actually be set to zero in the first-order model because viscous–thermal losses in water over centimeter-scale propagation distances is very small compared with amplitude reduction due to geometric spreading and repeated wall reflections. For example, according to Kinsler et al.[62], even assuming an attenuation of 0.02 dB/cm at MHz frequencies, a 2 cm propagation path corresponds to only 0.04 dB loss, which yields an amplitude factor of approximately 0.995, i.e., 0.5% reduction. In contrast, each wall reflection scales the pressure amplitude by the reflection coefficient Γ = 0.56, corresponding to a 44% reduction (≈ −5 dB). Therefore, bulk attenuation is negligible relative to boundary-induced amplitude reduction in the present configuration. It is noted that the presence of the bubble at the acoustic focus can locally perturb the pressure field due to reflection and transmission at the gas–liquid interface. Owing to the strong acoustic impedance mismatch between water and gas, only a small fraction of the incident pressure is transmitted into the bubble, while most of the wave is reflected at the interface. However, as shown in Fig. 2c, the radius of curvature of the converging shock front remains much larger than the bubble radius, so the incoming wave can be approximated as locally cylindrical at the bubble scale. In addition, the shock impact induces local flattening of the bubble interface, which further reduces curvature effects and promotes locally planar or cylindrical reflection behavior. Therefore, the bubble primarily modifies the local pressure loading, while the global focusing process and echo timing remain governed by the tube geometry.

Fig. 2a illustrates the simulated propagation of an acoustic shock wave in a cylindrical cuvette, employing COMSOL Multiphysics[63]. The source was generated by a monopolar mass source activated at *t* = 0. The screenshots show the spatial pressure distribution at successive times, with positive pressure indicated in red and negative (tensile) pressure in blue. After emission from the central source, the pressure wave propagated radially outward toward the cylindrical wall, where partial reflection occurred due to the acoustic impedance mismatch between water and the PMMA boundary. The reflected wave then refocused toward the center of the cylinder, forming a sequence of concentric pressure fronts that repeatedly traversed the central region. As shown in Fig. 2(*a*1-*a*4), the changes of wavefront size during propagation indicated only geometrical attenuation in the bulk liquid, while the reduced amplitude of the reflected fronts (figure 2*a5*) demonstrated that energy loss was dominated by partial transmission into the wall. In the last four frames of Fig. 2a, the dark blue region corresponds to the tensile (negative) pressure wave generated after reflection and subsequent focusing. The peak negative pressure in the horizontal plane increases from approximately − 2.5 MPa at t = 0.007 ms to about − 3.0 MPa at t = 0.010 ms, confirming that the tensile wave also undergoes geometric focusing toward the axis. However, the amplification of the compressive shock is stronger than that of the tensile wave. This difference is attributed to nonlinear steepening of compressive shocks during convergence, whereas rarefaction (tensile) waves tend to broaden and are additionally affected by refraction at the outer boundary. This visualization confirmed that successive echoes arose from geometric confinement and impedance-controlled reflections, supporting the modeling assumption that amplitude decay was governed primarily by wall reflections rather than viscous–thermal dissipation. Thus, each reflection reduced the pressure amplitude by the wall reflection factor Γ and the factor β representing imperfect re-focusing, so the k-th echo scales as

(4) $$p_{c}^{\left(k\right)}={\left(\Gamma \beta \right)}^{k}\;p_{0}\;\frac{r_{0}}{\Delta },\left(k=1,2,\cdots \right).$$

This formulation provides a simplified but physically consistent description of pressure amplification caused by cylindrical confinement and allows estimation of the relative strength of successive reflected shock-wave echoes reaching the bubble center. In the present work, Δ = 0.1 mm is used as a representative near-axis focusing scale. This order of magnitude is consistent with experimentally reported spatial pressure variation lengths in high-resolution shock and acoustic measurements in water[64]. Since the focusing factor scales as $r_{0}$, variations of Δ primarily rescale the predicted echo amplitudes but do not affect echo arrival times or the confinement-controlled trends discussed below. The arrival time of the k th reflected shock at the bubble center, denoted by $t_{c}^{\left(k\right)}$, corresponds to the round-trip propagation of the wave between the bubble and the tube wall. It may be expressed as

(5) $$t_{c}^{\left(k\right)}=\frac{2kR}{c}$$

Each subsequent echo thus reached the bubble at intervals of $\frac{2R}{c}$, corresponding to successive reflections of the initial shock wave between the confining walls.

Fig. 2b illustrates the radial pressure distribution of the propagating wave, showing the outward-propagating primary shock, its partial reflection at the tube wall (R = 8 mm), and the resulting inward-propagating shock together with the tensile rarefaction wave. To further assess the geometric character of the focusing shock, the radius of curvature of the wavefront near the tube axis was evaluated from the COMSOL numerical simulations. The resulting time evolution is shown in Fig. 2c. Throughout the propagation toward the axis, the curvature radius remains significantly larger than the characteristic bubble radius. Consequently, the incoming shock can be approximated as locally cylindrical. This confirms that the pressure loading experienced by the bubble arises from a cylindrically converging wave rather than from spherical focusing. The inset of Fig. 2c provides a three-dimensional visualization of the shock structure at t=9.5μs, shortly before the wave reaches the focal region on the axis. The geometry clearly illustrates the confined cylindrical focusing produced by reflections at the tube wall.

**Fig. 3:**
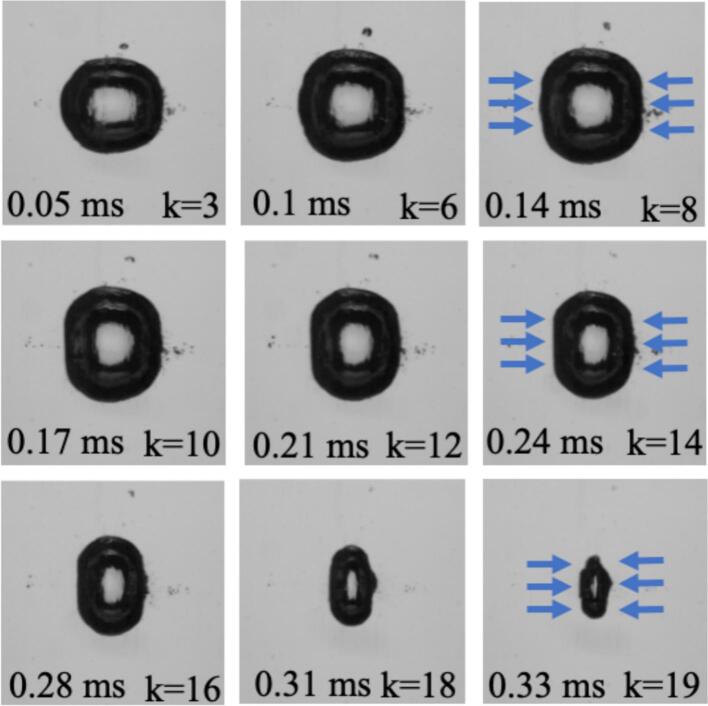
Time-resolved visualization of a laser-induced cavitation bubble confined within a cylindrical tube of radius R=13 mm and a maximum equivalent radius of $r_{eq,max}=1.5\;\text{mm}$. Each k denotes the number of acoustically reflected shock wave echoes that reached the bubble up to that moment. The arrows indicate the arrival of successive echo fronts, which compressed the bubble along the tube axis. The progressive prolate deformation and axial focusing observed at later stages were induced by the effect of the echo–bubble interactions.

Section 3.2 presents these analytical results, which provide the quantitative basis for the experimental observations. It is important to note that the echo model in this section is intended to capture the confinement-controlled timing and scaling of reflected pressure pulses. While a finite-size bubble locally scatters and partially reflects incoming pulses, its maximum diameter remains small compared to the tube diameter (e.g. ∼3 mm vs 26 mm in Fig. 3), corresponding to only about 1% of the tube cross-sectional area. Therefore, the wall-governed echo recurrence is not expected to change appreciably; the coupling mainly affects the effective pressure acting on the bubble interface rather than the global echo dynamics.

### 3.2 Effects of shock-wave echoes on bubble dynamics

The interaction between the reflected shock-wave echoes and the laser-induced cavitation bubble was experimentally investigated to assess how successive pressure pulses influenced bubble dynamics. High-speed imaging revealed that the returning echoes, which arrived within a few hundred microseconds after breakdown, significantly modified the collapse behavior and shape evolution of the bubble. Depending on their amplitude and timing relative to the bubble’s oscillation phase, the echoes may have enhanced collapse intensity, induced surface flattening, and promoted a transition to a prolate geometry. These observations demonstrated that even weak, delayed shock reflections could have played a decisive role in the asymmetric collapse of single bubbles confined within cylindrical boundaries. To generalize the results and remove the direct dependence on the laser input energy, an equivalent equilibrium radius $r_{eq}$ was defined that representee the characteristic bubble size. It was calculated from the instantaneous horizontal and vertical radii, $r_{h}$ and $r_{v}$, according to $r_{eq}^{3}=r_{h}^{2}r_{v},$ such that $r_{eq}$ corresponded to the radius of a spherical bubble having the same volume as the deformed one.

Fig. 3 presents the time-resolved sequence of a laser-induced cavitation bubble confined within a cylindrical tube of radius R=13 mm and a maximum equivalent radius of $r_{eq,max}=1.5$ mm. The sequence captured one complete oscillation period T, where each frame is identified by time t and its corresponding echo number k, denoting the count of reflected shock wave echoes that reached the bubble. As seen, the bubble remained nearly spherical during its early expansion (t=0.05ms, *k* = 3), but progressive vertical elongation became evident as successive echoes arrived. Between t=0.1 and 0.24 ms (k=6to 14), the lateral interfaces flattened due to compressive forcing from the reflected waves, consistent with the pressure focusing along the tube axis. This deformation intensified throughout the collapse phase (t=0.28to 0.33 ms), when up to 19 echoes interacted with the bubble, resulting in a strongly prolate geometry and an axially focused collapse. This sequence demonstrated that repeated, microsecond-spaced shock-wave reflections enhanced axial compression, providing direct visual evidence of echo-driven modulation and collapse anisotropy in confined cavitation. It is important to note that because the peak amplitude of successive echoes decreases rapidly with reflection order, their influence on bubble dynamics is governed primarily by the first few reflections, which retain sufficiently high amplitudes to interact with the bubble interface. At maximum expansion, the internal bubble pressure reaches a minimum and the interface becomes highly compliant. For a bubble of R = 1.5  mm, the surface-tension pressure is approximately 96 Pa. Using the representative parameters in Eq. (4) (p_0_ = 1 GPa, $r_{0}$=50  μm, Δ = 0.1 mm), the reflected echo pressure decays rapidly with reflection order. In practice, the first few echoes dominate the interaction, while higher-order reflections become progressively weaker and quickly fall below dynamically relevant pressure levels. Moreover, the influence of these early echoes is not strictly instantaneous: the pressure impulse delivered by the reflected shock accelerates the surrounding liquid, and due to hydrodynamic inertia the resulting interface motion can continue to evolve even after the shock wave itself has passed. As a result, deformation observed later in the collapse stage can originate from the impulse imparted by the first few echoes. Consistent with the experimental observations, bubble deformation and collapse morphology are therefore primarily governed by the early high-amplitude echoes rather than by the cumulative action of many weak reflections. Beyond several reflections (typically after k≈6), the reflected pressure becomes too small to significantly influence the bubble dynamics.

**Fig. 4:**
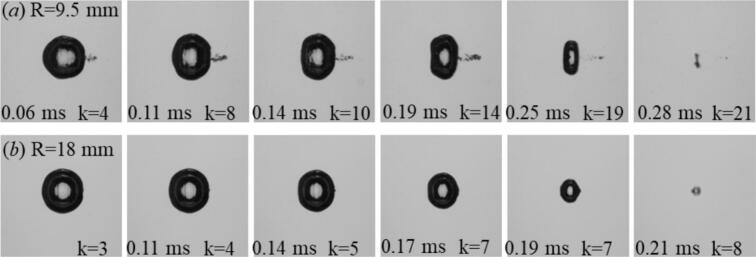
Comparative laser-induced bubble dynamics for the two tube radii of R=9.5 mm and R=18 mm at a constant maximum equivalent radius of $r_{eq,max}=1.1$ mm. Each frame shows the bubble morphology at selected times t, with k denoting the number of acoustically reflected shock-wave echoes that reached the bubble up to that instant. In the smaller tube, the shorter acoustic path causes the reflected echoes to return earlier, leading to stronger axial compression and pronounced prolate deformation during collapse. In contrast, the larger tube exhibits fewer echoes within one oscillation period, resulting in a nearly spherical collapse.

Fig. 4 compares the evolution of laser-induced cavitation bubbles with a maximum equivalent radius of $r_{eq,max}=1.1$ in two cylindrical tubes with inner radii of R = 9.5  mm and R = 18  mm. In the smaller tube, the shorter acoustic path causes the first high amplitude reflected echoes to return earlier. The early high-amplitude echoes compress the bubble along the tube axis and initiate a pronounced prolate deformation during collapse (t/T≈0.9). In contrast, the larger tube yields fewer echoes that arrive later and with reduced amplitude, leading to weaker axial compression and a less prolate-like collapse. This comparison highlights the key role of echo timing and amplitude in governing the echo–bubble interaction. Smaller confinement radii enhance acoustic feedback and promote asymmetric collapses, whereas larger geometries approach quasi–free-field behavior with more symmetric rebounds.

**Fig. 5:**
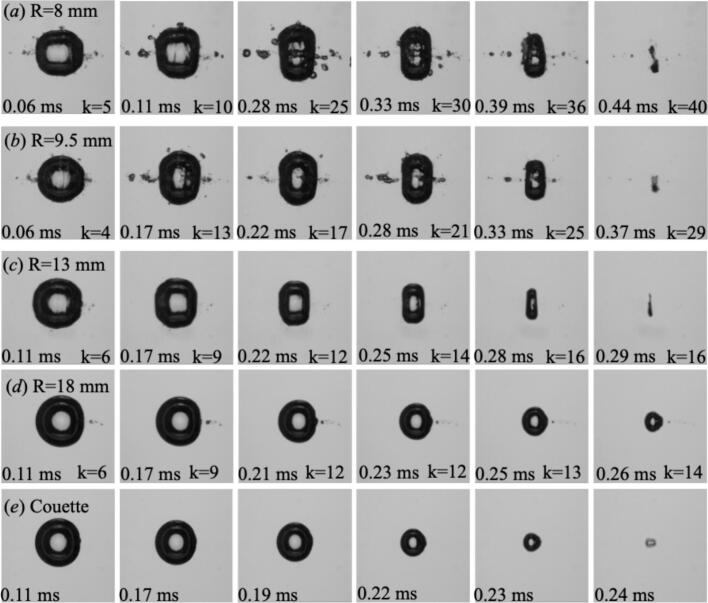
Comparative laser-induced bubble dynamics for different tube radii at a constant maximum equivalent radius of $r_{eq,max}=1.3$ mm. The sequences show the temporal evolution of the bubble within cylindrical tubes of (a) R=8 mm, (b) R=9.5 mm, and (c) R=13 mm, along with (d) a reference case in a Couette cell representing an unconfined, weakly reflecting condition. As the tube radius decreased, the echo frequency acoustic forcing increased, leading to stronger axial compression and more pronounced prolate deformation during collapse. In contrast, the Couette case shows a nearly spherical evolution with minimal echo influence, demonstrating the pivotal role of confinement-induced reflections in shaping bubble dynamics.

Fig. 5 compares the evolution of cavitation bubbles with a fixed maximum equivalent radius of $r_{eq,max}=1.3$ mm generated in cylindrical tubes of different radii (R=8, 9.5, and 13 mm) and in a reference Couette configuration representing a weakly reflective environment. In the smallest tube (R=8 mm), the short acoustic path resulted in rapid and higher echo frequency of up to 94 kHz. As the tube radius increased to R=9.5 mm, the echoes arrived at less frequency (∼79 kHz). For the largest tube (R=18 mm), the echo frequency reduces to about 42 kHz, and the bubble remained nearly axisymmetric, indicating weaker forcing. The Couette case, in which reflections were minimal and not focal, exhibited a fully symmetric collapse consistent with free-field dynamics. This systematic variation with R demonstrated that the degree of echo-driven deformation was primarily governed by the confinement geometry: Smaller radii produced higher echo frequencies and greater pressure reinforcement, whereas larger or weakly reflective boundaries suppressed echo coupling and preserved the near-spherical collapse behavior.

**Fig. 6:**
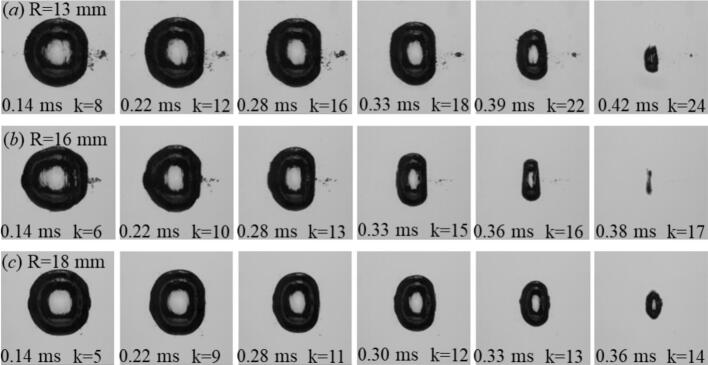
Comparative cavitation bubble dynamics for three tube radii (R=13, 16, and 18 mm) at a constant maximum equivalent radius of $r_{eq,max}=1.7$ mm. In narrower tubes (R=13 mm), the echoes arrived more frequently, resulting in stronger axial compression and pronounced prolate collapse. With increasing tube radius, the echo return time became longer and fewer reflections occurred within one oscillation period, leading to weaker forcing and a more symmetric collapse.

Fig. 6 illustrates the influence of tube radius on bubble dynamics for a higher driving condition, corresponding to a maximum equivalent radius of $r_{eq,max}=1.7$ mm. Compared with the smaller bubble cases shown above, the oscillation period here is longer, allowing a larger number of reflected echoes to reach the bubble during its lifetime. In the most confined geometry (R = 13  mm), the echo frequency reaches approximately 58 kHz. As the tube radius increases to R = 16  mm, the echo frequency decreases to about 47 kHz and the resulting deformation becomes less pronounced, although the collapse remains asymmetric. In the widest tube (R = 18  mm), the bubble approaches a nearly axisymmetric collapse. These observations indicate that bubble deformation is primarily governed by the timing and amplitude of the earliest reflected echoes. Smaller tube radii cause the first echoes to return earlier and with higher amplitude, producing stronger axial compression and more pronounced prolate deformation, whereas larger tubes delay and weaken the early reflections, leading to more symmetric collapse behavior.

**Fig. 7:**
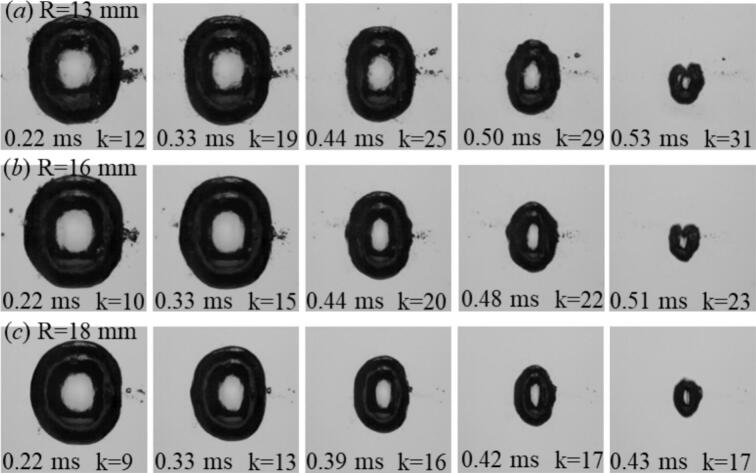
Comparative cavitation bubble dynamics for three tube radii (R=13, 16, and 18 mm) at a maximum equivalent radius of $r_{eq,max}=2.0$ mm. The extended oscillation period associated with this large bubble size allowed strongest and numerous echoes to interact with the bubble during its lifetime. In all cases, the acoustic forcing induced a pronounced prolate deformation.

Fig. 7 presents the evolution of cavitation bubbles with a maximum equivalent radius of $r_{eq,max}=2.0$ mm, representing the strongest driving condition investigated. At this large scale, both the initial shock-wave amplitude and the oscillation period were significantly increased, allowing reflected shock waves to interact with the bubble over an extended time interval during its expansion and collapse phases. Even in the widest tube (*R = 18 mm*), the early reflected echoes retained sufficient amplitude to influence the bubble dynamics, maintaining an elongated, prolate morphology during much of the oscillation cycle. In the most confined case (*R = 13* mm), the shorter acoustic path resulted in a higher echo frequency, leading to stronger and earlier axial compression of the bubble. As the tube radius increased to *R = 16 mm*, the echo frequency decreased, and the deformation became less pronounced. In the widest tube (*R = 18 mm*, the echo frequency further decreased and the collapse approached a more axisymmetric behavior. These results demonstrate that bubble deformation is governed primarily by the timing and amplitude of the earliest reflected echoes. The experimental trends are consistent with the echo-pressure model introduced in Section 3.1 (Fig. 2a), in which the amplitude and recurrence of echoes at the bubble center are determined by the initial shock strength $P_{0}\left(r_{\mathrm{eq}}\right)$ and the geometric confinement radius *R*. Increasing the equivalent radius $r_{\mathrm{eq}}$ corresponds to stronger laser input, producing larger bubbles with longer oscillation periods and stronger primary shocks. Consequently, bubbles with larger $r_{\mathrm{eq}}$ experience stronger reflected echoes over a longer interaction time, while the tube radius controls the echo frequency and therefore the timing of the echo–bubble interaction. The combined effects of echo attenuation (Eq. (2) and geometry-controlled echo timing provide a coherent physical framework for the experimentally observed size- and confinement-dependent modulation of bubble dynamics.

**Fig. 8:**
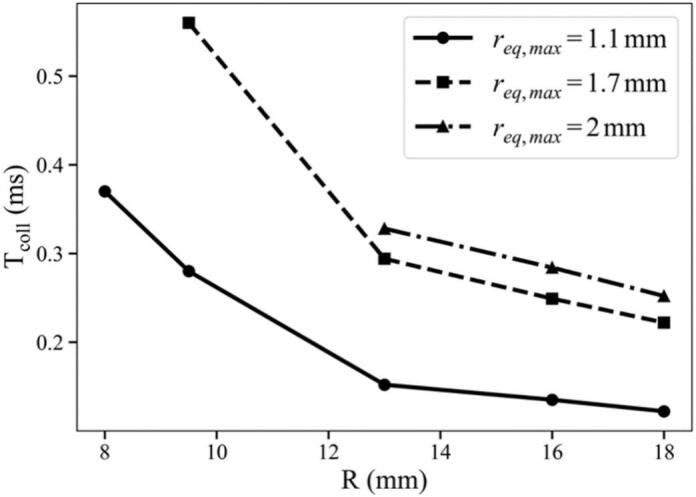
Variation of the bubble collapse time $T_{coll}$ as a function of the tube radius R for different maximum equivalent radii $r_{eq}$ . The collapse time decreases with increasing R as the confinement effect weakens in larger tubes. It also increases with $r_{eq,max}$ , since larger bubbles exhibit longer oscillation periods.

Fig. 8 plots the variation of the collapse time $T_{coll}$ as a function of the tube radius R for different maximum equivalent radii $r_{eq,max}$. The results show that $T_{coll}$ increases as the tube radius decreases, indicating a stronger confinement effect in smaller tubes. This trend is consistent with previous studies of bubbles in confined geometries, such as the work of Yuan et al.[47], who reported that channel confinement can increase bubble lifetime. Larger bubbles also exhibit longer collapse times due to their longer oscillation periods. Simple calculations of imploding empty bubbles in a tube, employing Klotz and Hyninen’s formulation[46], and independently OpenFOAM[65], [66], could roughly reproduce the observed expansion of $T_{coll}$ for the larger cylinder radii. As expected from Rayleigh-type inertial scaling, $T_{coll}$ increases with $r_{\mathrm{eq}}$ because larger bubbles oscillate over longer time scales. The longer lifetime then allows a greater number of reflected pulses to interact with the bubble. Echoes primarily modulate the collapse dynamics and symmetry, and can additionally shift $T_{coll}$ depending on their phase. The additional increase of collapse time in smaller tubes is attributed to repeated echo forcing and the associated departure from spherical collapse. As the tube radius decreases, the echo round-trip time becomes shorter and a larger number of pressure pulses interact with the bubble within one oscillation. These echoes intermittently perturb the interface acceleration during late expansion and collapse, effectively slowing the monotonic collapse. Moreover, the higher repetition rate promotes stronger prolate deformation; once the collapse becomes non-spherical, part of the collapse energy may be diverted into shape modes and axial flow rather than purely radial implosion, which further prolongs the time to reach minimum volume. Thus, the longer bubble lifetime in smaller tubes probably results from the combined effects of confinement, more frequent phase-dependent echo forcing and deformation-induced reduction of collapse efficiency. In contrast, for wider tubes, the echoes arrived less frequently, and the bubble experienced weaker reinforcement. Overall, the results confirmed that both the lifetime and deformation of the bubble were dictated by the interplay between its equilibrium size and the confinement geometry governing echo timing and attenuation.

**Fig. 9:**
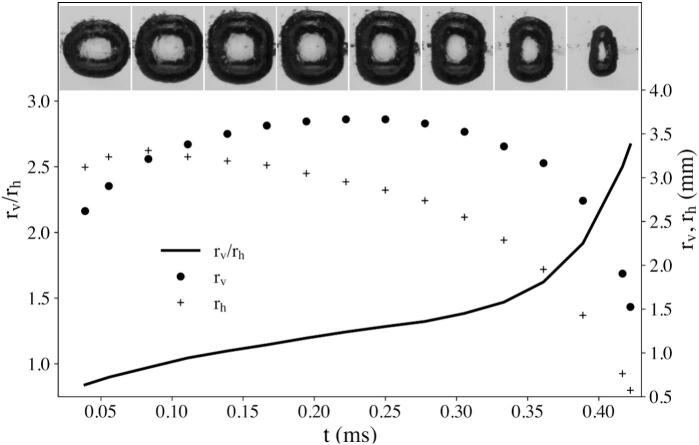
Temporal evolution of the vertical radius $r_{v}$, the horizontal radius $r_{h}$, and the corresponding aspect ratio $r_{v}/r_{h}$ for the case R=13 mm and $r_{eq,max}=1.8$ mm. During the early expansion phase (t<0.1 ms), the bubble remained nearly spherical, but as successive echoes arrived, the vertical radius $r_{v}$ grew faster than the horizontal radius $r_{h}$, causing $r_{v}/r_{h}$ to increase progressively. Near the final collapse stage (t≈0.4 ms), the ratio rose sharply. The top image sequence illustrates the corresponding bubble morphologies at representative time instants.

Fig. 9 presents the time-resolved evolution of the bubble geometry for the case R=13 mm and $r_{eq,max}=1.8\;$ mm, representing a strongly driven condition in which echo interactions were particularly pronounced. During the early expansion stage (t<0.1 ms), the aspect ratio remained close to unity, indicating an initially spherical bubble. As the oscillation proceeded, the vertical axis $r_{v}$ grew faster than $r_{h}$, and the ratio $r_{v}/r_{h}$ rose to about 3.5, signifying progressive elongation along the tube axis caused by successive echo-induced compressions. In the late collapse phase (t≈0.4ms), $r_{h}$ and $r_{v}$ decreased sharply, while $r_{v}$ remained relatively larger, leading to a steep increase in $r_{v}/r_{h}$ that marked the onset of the final prolate collapse.

**Fig. 10:**
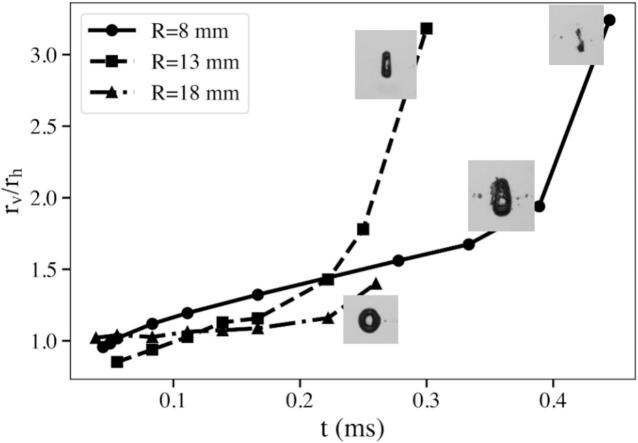
Temporal evolution of the bubble aspect ratio $r_{v}/r_{h}$ for different tube radii (R=8, 13 and 18 mm) at a maximum equivalent radius of $r_{eq,max}=1.3$ mm. In the most confined case (R=8 mm), the high echo frequency resulted in a gradual, but sustained elongation throughout the oscillation and the delayed collapse. For R=13 mm, although fewer echoes arrived, their impact coincided with the collapse phase, producing a rapid increase in $r_{v}/r_{h}$ and a strongly prolate final shape. In contrast, for the widest tube (R=18 mm), the echoes arrived less frequently, leading to smaller overall deformation and a nearly symmetric collapse.

Fig. 10 compares the time evolution of the bubble aspect ratio $r_{v}/r_{h}$ for different tube radii at $r_{eq,max}=1.3$ mm. The results show how the interplay between echo frequency and bubble collapse timing governed the degree of axial deformation. In the smallest tube (R=8 mm), the short round trip path caused echoes to return at high frequency, resulting in elongation and delayed collapse. For the intermediate case (R=13 mm), the collapse time $T_{coll}$ was shorter, so the arriving echoes coincided with the most compressive phase of the bubble motion. These high-intensity, well-timed echoes amplified the axial acceleration and caused a sharp rise in $r_{v}/r_{h}$, indicating a strong prolate collapse. In the largest tube (R=1 8 mm), the echo frequency was much lower, reducing the ability to reinforce the collapse; consequently, the aspect ratio remained small and the bubble collapse was nearly symmetric. Overall, these results demonstrated that the magnitude and timing of echo arrivals, controlled by the tube radius, determined whether deformation built up gradually or culminated abruptly during the collapse phase.

**Fig. 11:**
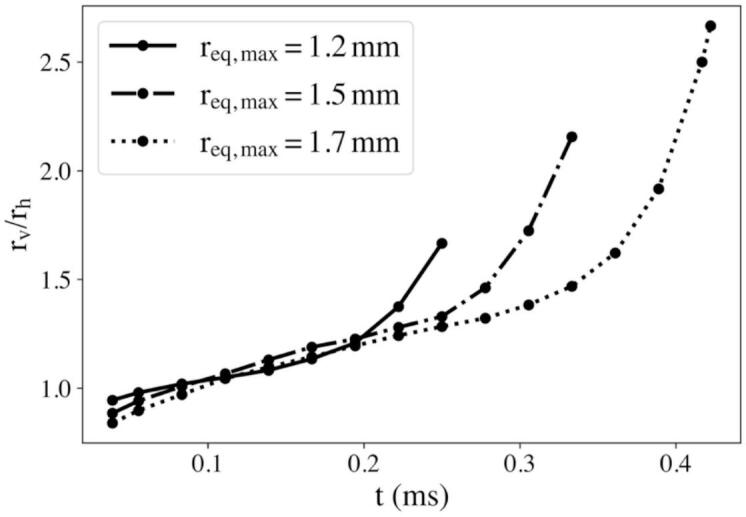
Temporal evolution of the aspect ratio $r_{v}/r_{h}$ for different maximum equivalent radii ($r_{eq,max}=1.2$, 1.5, and 1.7 mm) in a tube of radius R=13 mm. Larger $r_{eq}$ values corresponded to stronger laser-induced shocks and longer collapse periods, allowing more and stronger echo interactions to occur before collapse. Consequently, the aspect ratio increased for larger $r_{eq}$, indicating enhanced axial deformation and a transition toward a prolate collapse shape.

Fig. 11 shows how the bubble aspect ratio $r_{v}/r_{h}$ evolved over time for different equilibrium radii $r_{eq}$ at a fixed confinement of R=13 mm. During the early expansion phase (t<0.1 mm), all cases remained nearly spherical, with $r_{v}/r_{h}\approx 1$. As the oscillation proceeded, the influence of repeated echo impacts became more pronounced. For larger $r_{eq}$, the echoes’ amplitude and bubble lifetime extended and the echoes had more opportunities to interact with the bubble surface before collapse. This forcing enhanced the vertical expansion rate, leading to a progressively steeper rise in $r_{v}/r_{h}$. At $r_{eq,max}=1.7$ mm, the aspect ratio exceeded 2.5 near the end of the cycle, reflecting strong echo-driven prolate deformation. In contrast, smaller bubbles ($r_{eq,max}=1.2$ mm) underwent fewer and weaker echo interactions and collapsed more symmetrically.

### 3.3 Effects of shock wave echoes on nano bubble dynamics

To complement the experimental observations presented above, molecular dynamics (MD) simulations were carried out to investigate the effects of shock waves on nanobubble dynamics at the atomic scale. The MD framework provided direct visualization of plasma-shock propagation, rebound behavior, and nanobubble deformation under conditions analogous to the confined laser-induced cavitation experiments. In the simulations, a focused laser pulse was directed toward the center of water confined within a cylindrical carbon nanotube (CNT), following the experimental geometry described above. To examine the influence of shock-wave intensity on bubble behavior, two laser energy levels of 1 and 10 fJ were considered. For each case, the system was first equilibrated to achieve thermodynamic stability before applying the laser energy to the designated radiation region using the laser-induced cavitation algorithm[53]. Separate simulations were performed for both energy levels using 1,120 processors on the amplitUDE supercomputer at the University of Duisburg-Essen. A detailed description of the computational model, including the system configuration, interaction potentials, and laser–cavitation algorithm, is provided in the Appendix.

**Fig. 12:**
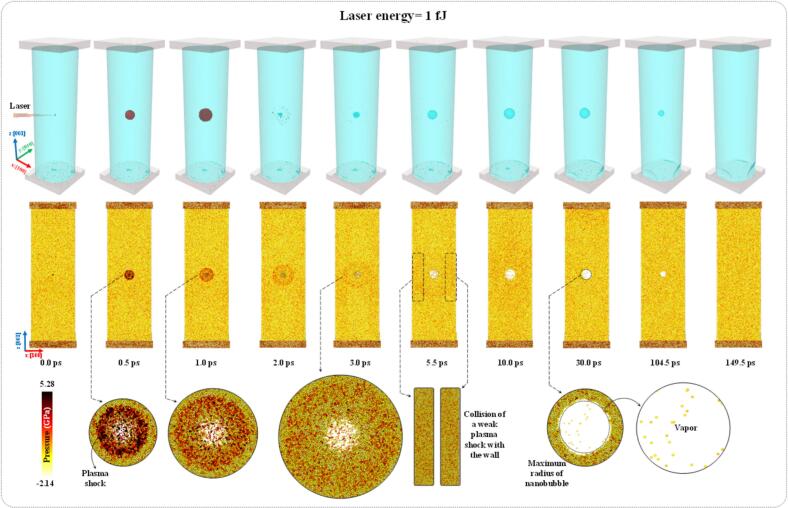
Surface mesh views and a pressure map of the system under 1 fJ laser irradiation. Upon laser exposure, a plasma shock formed and propagated through the medium. Subsequently, a nanobubble nucleated, grew, and eventually collapsed. The vertical color bar to the left of the surface mesh views identifies pressure magnitudes in the cross-sectional z–x plane of 1 mm thickness.

**Fig. 13:**
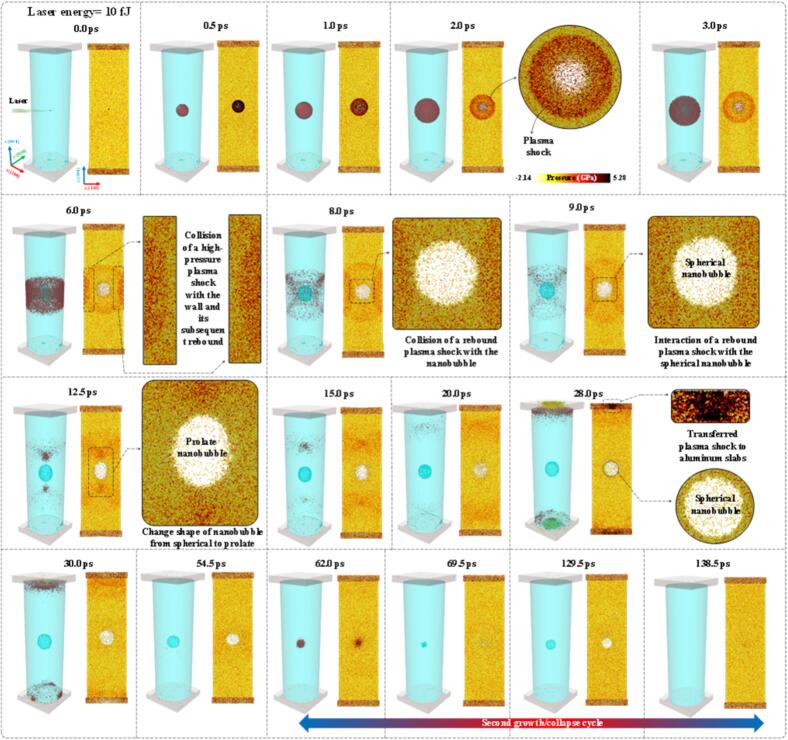
The surface mesh view and pressure map of the system under 10 fJ laser irradiation. Upon laser exposure, a plasma shock formed and propagated through the medium. Subsequently, a nanobubble nucleated, grew, and eventually collapsed. The horizontal color bar beneath the surface mesh view at 2.0 ps identifies pressure magnitudes in the cross-sectional z–x plane of 1 mm thickness.

Fig. 12, Fig. 13 show successive screenshots from simulations corresponding to laser energies of 1 and 10 fJ, respectively. Fig. 12 presents side-by-side screenshots of the three- and two-dimensional cases. The three-dimensional screenshots provide a surface-mesh representation of system, illustrating the plasma shock wave, its propagation, its interactions with the CNT wall, the nanobubble, and the aluminum slab as well as the overall nanobubble dynamics. In contrast, the two-dimensional screenshots display also pressure contours in the cross-sectional z–x plane with a thickness of 1 nm, corresponding to the surface-mesh views. This figure demonstrated that, when a laser with an energy of 1 fJ was irradiated at the center of the water, a high-temperature and high-pressure region formed, resembling a plasma state. The excited water molecules within the irradiated zone had high kinetic energy and tended to move apart. These energized molecules collided with surrounding water molecules, transferring part of their energy through successive molecular collisions, which led to a cascade effect. Consequently, a plasma shock wave propagated spherically outward from the center, as illustrated by the red spherical mesh in Fig. 12. At the moment of laser irradiation, the temperature and pressure of water molecules within the plasma shock exceeded 5000 K and 290 GPa, respectively. However, over time, these values decreased rapidly as the shock transferred energy to the surrounding medium.

Tracking the plasma shock over time revealed that, following laser irradiation, a spherical plasma shock propagated and reached the CNT wall after about 5.5 ps (Fig. 12). Considering that the distance between the center of the cylinder and its wall was 24.9 nm, the average velocity of the plasma shock was estimated to be 4.5 km/s, which agreed well with previously reported experimental data that indicated plasma propagation velocities in the range of 3 to 7 km/s [67], [68]. After 5.5 ps, the plasma shock impacted the cylinder wall, its momentum was reversed due to the rigid boundary effect and, subsequently, it propagated in the opposite direction. However, because of its limited energy and momentum, the rebounded shock dissipated after several picoseconds and failed to interact with the nanobubble wall. As a result, the nanobubble underwent spherical growth and collapse, consistent with the experimental observations reported above under low laser energy conditions.

Based on Fig. 12, it was apparent that during the first 2.0 ps of plasma shock propagation, the water molecules were displaced outward from the center, creating the necessary conditions for nanobubble formation. As a result, a nanobubble nucleus appeared after about 2.0 ps. Within this nucleus, high-pressure and high-temperature water molecules exerted outward forces that expanded the bubble. Simultaneously, the plasma shock continued to push the surrounding water molecules outward, causing the nanobubble to grow and reach its maximum radius after about 30.0 ps. At this stage, the vapor phase inside the nanobubble was clearly observed. Subsequently, the external pressure exceeded the internal vapor pressure, leading to nanobubble collapse, which was completed after about 149.5 ps. After this collapse, the residual energy at the collapse center was insufficient to trigger a secondary regrowth/collapse cycle. The nanobubble generated by a laser energy of 1 fJ exhibited a maximum radius of 6.2 nm and lasted for a lifetime of 147.5 ps.

Fig. 13 presents the screenshots of the surface mesh and pressure contour following laser irradiation with an energy of 10 fJ. The results indicated that, under laser irradiation with ten times higher energy, the overall phenomena of plasma shock formation and propagation remained qualitatively similar to the 1 fJ case. The plasma shock formed and propagated spherically through a cascade of molecular collisions, leading to the nucleation of a nanobubble after about 2.0 ps. However, due to the higher laser energy, a stronger plasma shock was generated and propagated more intensely compared to the 1 fJ case. The pressure map revealed that the plasma shock front was associated with a larger number of excited water molecules experiencing pressures exceeding 4 GPa. The plasma shock reached the CNT wall after 4.1 ps, corresponding to a propagation velocity of about 6 km/s. A comparison of propagation velocities between the 1 fJ and 10 fJ cases demonstrated that increasing the laser energy by a factor of ten while maintaining a constant impact cross-section, resulted in an approximate 33.3% increase in plasma shock velocity.

When the plasma shock impacted the cylinder wall, the momentum of the water molecules at the shock front was reversed, generating a semi-spherical rebound shock that propagated in the opposite direction, as seen in Fig. 13 at 6.0 ps. Meanwhile, other portions of the plasma shock traveling along the cylinder axis continued to propagate outward. The rebound shock progressed toward the center and reached the nanobubble after about 8.0 ps. During this period, the nanobubble expanded spherically, attaining a maximum radius of 10.5 nm. When the rebound shock interacted with the nanobubble wall after 8.0 ps, the vapor pressure inside the bubble counteracted the incoming shock pressure. However, this interaction compressed the nanobubble and generated a shock-induced flow along its surface in the axial direction. Consequently, the nanobubble deformed from a spherical to a prolate shape, with radii of 8.7 and 11.2 nm, respectively, observed after 12.5 ps. These findings are consistent with experimental observations showing that, upon interaction with rebound shocks, the initially spherical shape of millimeter-scale bubbles transitioned to a prolate form.

The cavitation nanobubble changes shape immediately after the arrival of the first reflected shock wave. The timing of this interaction is governed by the acoustic round-trip distance between the bubble center and the cylinder wall. In the nanoscale cylinder (diameter ≈ 49.8 nm), the reflected shock returns within a much shorter time than in the millimeter-scale experiment. Since the first reflection carries the highest amplitude, it dominates the deformation process. If the cylinder diameter were varied, the arrival time would shift accordingly, and the bubble could be in a different dynamic stage (growth, maximum radius, or collapse) at the moment of impact, resulting in different deformation intensity. After deformation, the nanobubble gradually returns to a spherical shape due to surface-tension-driven relaxation. According to the Young–Laplace relation, the pressure difference across the interface scales with 1/R; therefore, the much smaller bubble radius at the nanoscale leads to significantly higher Laplace pressures and faster restoration of sphericity compared with the millimeter-scale case. Due to the computational expense of the MD simulations, it was not feasible to extend the cylinder radius, bubble size, or simulation time significantly. Larger nanoscale systems and longer time windows would likely allow observation of additional reflected shock interactions, analogous to the multi-echo effects observed in the macroscopic experiments. However, within the present computationally accessible scale, the first reflected shock dominates the deformation process.

After the nanobubble changed its shape from spherical to prolate (Fig. 13 after 12.5 ps), the rebound shock propagated upward and downward along the cylinder axis, colliding with the aluminum slabs and transferring momentum to these holders. Subsequently, a portion of the plasma shock rebounded from the aluminum slab (Fig. 13 after 30.0 ps) and propagated toward the nanobubble, colliding with it and inducing the first collapse after 61.0 ps. During this initial growth/collapse cycle, the nanobubble reached a maximum radius of 8.7 nm and a radius of 11.2 nm in its prolate shape, with a lifetime of 59.0 ps. Upon the first collapse, water molecules returning toward the bubble center collided with one another, generating a high-temperature and high-pressure region that subsequently triggered a second regrowth/collapse cycle through a cascade of collisions. In the second cycle, the nanobubble expanded spherically to a maximum radius of 3.4 nm, with a lifetime of 76.5 ps.

As an interface among the experimental results and MD data, it can be inferred that both exhibit a transition from spherical to prolate bubble shape following the arrival of a reflected shock wave. The COMSOL simulations confirm the geometric refocusing of the shock toward the tube axis, while the MD simulations provide atomistic visualization of the subsequent bubble–shock interaction. Although the macroscopic and nanoscale systems differ significantly in absolute size, the deformation mechanism is governed in both cases by anisotropic pressure loading induced by the reflected shock wave. The prolate deformation results from these pressure gradients rather than from classical inertial–capillary balance. Therefore, the consistency between experiment and MD simulation reflects agreement in the dominant shock-driven deformation mechanism, rather than strict similarity of conventional dimensionless numbers across scales.

## 4 Conclusions

This study investigated the interaction between acoustically reflected shock-wave echoes and laser-induced cavitation bubbles confined within cylindrical tubes. The experiments demonstrated that the timing and amplitude of the reflected shocks, governed by the tube radius, play a decisive role in shaping bubble dynamics. The earliest reflected echoes, returning within microseconds after the initial laser breakdown, strongly influence the collapse behavior by compressing the bubble along the tube axis. These early high-amplitude echoes initiate pronounced prolate deformations during collapse. Systematic variation of the confinement geometry revealed that smaller tube radii enhanced echo frequency due to shorter acoustic paths, while larger tubes delay the echoes, resulting in bubble dynamics that approach quasi–free-field behaviour with nearly spherical collapses. To complement the experiments, MD simulations were performed to explore the effects of plasma-induced shock waves on nanobubble dynamics under a cylindrical confinement. At low laser energy (1 fJ), the generated plasma shock lacked sufficient strength to rebound from the wall, resulting in the formation and symmetric collapse of a spherical nanobubble with a maximum radius of 6.2 nm and a lifetime of 147.5 ps. In contrast, at higher energy (10 fJ), a stronger rebound shock propagated through the confinement and interacted with the nanobubble, producing a transition from a spherical to a prolate shape with radii of 8.7 and 11.2 nm and a lifetime of 59.0 ps. The prolate nanobubble subsequently relaxed back to a spherical configuration due to the restoring Young-Laplace pressure.

Overall, our findings established a unified physical picture of echo-driven cavitation in confined geometries, showing that boundary-induced acoustic reflections acted as a secondary driving mechanism for asymmetric bubble collapse. For future work, it should be of particular interest to examine how the pressure reflection coefficient Γ at the wall-liquid interface influences echo-driven bubble dynamics. In our present study, the PMMA walls provided a moderate reflection coefficient of Γ=0.37, resulting in partially transmitted and partially reflected pressure waves. Materials with a higher Γ should generate stronger reflected shocks and thus more intense echo sequences, potentially amplifying bubble dynamics.

## CRediT authorship contribution statement

**Mazyar Dawoodian:** Writing – review & editing, Writing – original draft, Visualization, Validation, Methodology, Investigation, Formal analysis, Conceptualization. **Sasan Rezaee:** Writing – review & editing, Visualization, Validation, Software, Investigation. **Dipanjan Barman:** Investigation. **Ould el Moctar:** Writing – review & editing, Supervision, Project administration, Methodology, Funding acquisition, Formal analysis, Conceptualization. **Rafael Manso Sainz:** Writing – review & editing, Visualization, Software, Investigation. **Robert Mettin:** Writing – review & editing, Methodology, Formal analysis, Conceptualization. **Christiane Lechner:** Writing – review & editing, Methodology.

## Declaration of competing interest

The authors declare that they have no known competing financial interests or personal relationships that could have appeared to influence the work reported in this paper.

## Figures and Tables

**Fig. A1:**
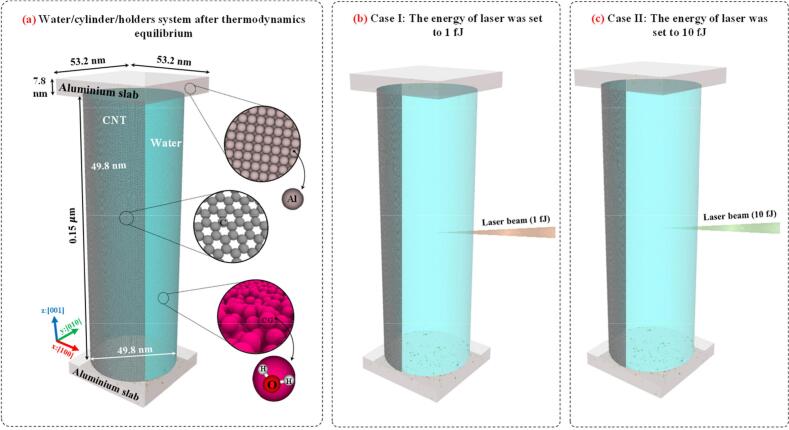
The atomic water/cylinder/holder system (a) after reaching its thermodynamic equilibrium state at 298 K, (b) at the instant of laser radiation with energy of 1 fJ (case I), and (c) at instant of laser radiation with energy of 10 fJ (case II).

## Appendix A Molecular dynamics, a computational method to simulate a nanobubble

### A1. Numerical modeling of the water/cylinder/holder system

To model the water/cylinder/holder system, a combination of visual molecular dynamics (VMD) [69], the large-scale atomic/molecular massively parallel simulator (LAMMPS) platform [70], and Packmol [71] packages were utilized. First, a carbon nanotube (CNT) with a chiral index of n = 368 and m = 368, a radius of 49.8 nm, and a length of 0.15 μm was constructed using VMD, and the resulting structure was saved in a Protein Data Bank (PDB) format. Next, an aluminum slab with dimensions of 53.2 × 53.2 × 7.8 nm^3^ was modeled in LAMMPS by replicating its face-centered cubic (FCC) lattice, and its data were also saved in PDB format. Subsequently, water molecules were modeled using a coarse-grained (CG) approach [53], [72], in which each water molecule was represented as a spherical bead interacting with the other molecules as an H_2_O unit. The CG model was defined using a custom script and saved in PDB format. All structures, including the CNT, the two aluminum slabs, and the CG water beads, were then imported into Packmol. Using Packmol, the CG water molecules were packed into the interior of the CNT, while the two aluminum slabs were positioned at the top and bottom of the CNT. As a result, the final configuration (Fig. A1a) consisted of a CNT that served as the cylindrical confinement, water at 0.997 g/cm^3^ that containing 9,737,592 molecules, and two aluminum slabs that served as system holders.

### A2. Interaction potential

The system illustrated in Fig. A1 consisted of three independent components: water, a CNT, and two aluminum slabs. The atomic interactions in the water medium were defined using a CG model. The CG model provided the necessary conditions for simulating large systems required for cavitation studies that accurately predicted water properties and cavitation phenomena [53], [72]. However, this model did not account for hydrogen bonding or the associated dipole moment of water molecules. The interactions between carbon atoms in the CNT wall were defined using the adaptive intermolecular reactive empirical bond-order (AIREBO) potential[73], which effectively reproduced the physical behavior of carbon allotropes. For the aluminum slab, the embedded atom method (EAM) potential [74] was employed, as it reliably predicted the metallic properties and interfacial behavior of aluminum in contact with water [75]. The van der Waals interactions between carbon atoms, the CG water beads, and the aluminum atoms were modeled using the Lennard-Jones 12–6 potential, with parameters adopted from previous studies [72], [76], [77] utilizing the Lorentz-Berthelot mixing rules [78].

### A3. Laser induced nano-cavitation algorithm

All molecular dynamics (MD) simulations were performed using the LAMMPS platform [70], and all visualizations were generated using the open visualization tool (OVITO) software [79]. Origin 2023 (Origin Lab, Northampton, MA, USA) was used to plot the diagrams and curves. For the simulations, the system was positioned at the center of a simulation box with dimensions of 53.3 × 53.3 × 166.1 nm^3^, and fixed boundary conditions were applied in all directions on the box walls to ensure the simulation of a single system. The Newtonian equations of motion were solved using the velocity-Verlet algorithm [80]. To induce nano-cavitation, the novel atomic laser algorithm for laser-induced cavitation bubbles, developed by Rezaee et al. in 2025 [53], was employed, as described below.

For the simulation, the CNT was fixed throughout the entire process to replicate the effect of a rigid cylindrical wall, as used in the experimental setup described above. Initially, the water was relaxed using a combination of the Langevin thermostat and the microcanonical (NVE) ensemble at 1 K [75], while the aluminum slab was excluded from integration. This stage was conducted with a time step of 0.05 fs (fs) over a 0.5 picosecond (ps) time window. Subsequently, the water reached thermodynamic equilibrium at 298 K under the canonical NVT ensemble over 1.0 ns (ns), with the aluminum slab still isolated from integration. In this stage, the time step was set to 1.0 fs. Thereafter, the aluminum slab was equilibrated under the NVT ensemble at 298 K for 1.0 ns, with water excluded from the integration. At this point, the system was fully equilibrated and prepared for laser exposure.

To apply laser energy to the water, a spherical region with a radius of 0.8 nm, containing 77 CG water beads, was selected at the center of the cylinder along its axis. The laser energy was then applied to this region using the laser algorithm for bubble cavitation. Detailed information regarding the mechanism of laser radiation in water, its role in inducing cavitation, and the associated plasma shock formation can be found elsewhere ^53^. After laser irradiation, the simulation was continued under the NVE ensemble to investigate the nano-cavitation under laser radiation as well as the formation and propagation of the resulting plasma shock. As the laser energy generated a rapidly propagating plasma shock, the timestep was reduced to 0.1 fs to ensure accurate time integration and proper tracking of atomic trajectories, positions, and velocities. The simulation was conducted for 1.0 ns, after which the trajectories and the thermodynamic parameters were analyzed to visualize the nano-cavitation process and the effects of the shock wave.

## References

[1] Lauterborn W., Vogel A. (2013). Shock wave emission by laser generated bubbles. Bubble Dyn. Shock Waves.

[2] Mason T.J. (2016). Ultrasonic cleaning: an historical perspective. Ultrason. Sonochem..

[3] Johnsen E., Colonius T. (2008). Shock-induced collapse of a gas bubble in shockwave lithotripsy. J. Acoust. Soc. Am..

[4] Ohl, S. W., Klaseboer, E. & Khoo, B. C. Bubbles with shock waves and ultrasound: A review. *Interface Focus* vol. 5 Preprint at https://doi.org/10.1098/rsfs.2015.0019 (2015). 10.1098/rsfs.2015.0019 PMC4549845 26442143

[5] Apfel R.E. (1982). Acoustic cavitation: a possible consequence of biomedical uses of ultrasound. Br. J. Cancer.

[6] Sackmann M. (1988). Shock-wave lithotripsy of gallbladder stones. N. Engl. J. Med..

[7] Delius M., Adams G. (1999). Shock wave permeabilization with ribosome inactivating proteins: a new approach to tumor therapy. Cancer Res..

[8] Ohl C.D., Ikink R. (2003). Shock-wave-induced jetting of micron-size bubbles. Phys. Rev. Lett..

[9] Dear J.P., Field J.E., Walton A.J. (1988). Gas compression and jet formation in cavities collapsed by a shock wave. Nature.

[10] Kodama T., Takayama K. (1998). Dynamic behavior of bubbles during extracorporeal shock-wave lithotripsy. Ultrasound Med. Biol..

[11] Wolfrum B., Kurz T., Mettin R., Lauterborn W. (2003). Shock wave induced interaction of microbubbles and boundaries. Phys. Fluids.

[12] Bokman G.T. (2025). Shock-induced bubble jets: a dual perspective of bubble collapse and interfacial instability theory. J. Fluid Mech..

[13] Freund J.B., Shukla R.K., Evan A.P. (2009). Shock-induced bubble jetting into a viscous fluid with application to tissue injury in shock-wave lithotripsy. J. Acoust. Soc. Am..

[14] Shima A., Tomita Y., Takahashi K. (1984). The collapse of a gas bubble near a solid wall by a shock wave and the induced impulsive pressure. Proc. Inst. Mech. Eng. C J. Mech. Eng. Sci..

[15] Lauterborn W., Bolle H. (1975). Experimental investigations of cavitation-bubble collapse in the neighbourhood of a solid boundary. J. Fluid Mech..

[16] Bourne N.K., Field J.E. (1991). Bubble collapse and the initiation of explosion. Proc. R. Soc. Lond. A Math. Phys. Sci..

[17] Sankin G.N., Simmons W.N., Zhu S.L., Zhong P. (2005). Shock wave interaction with laser-generated single bubbles. Phys. Rev. Lett..

[18] Bokman G.T., Biasiori-Poulanges L., Meyer D.W., Supponen O. (2023). Scaling laws for bubble collapse driven by an impulsive shock wave. J. Fluid Mech..

[19] Coussios C.C., Roy R.A. (2008). Applications of acoustics and cavitation to noninvasive therapy and drug delivery. Annu. Rev. Fluid Mech..

[20] Coleman A.J., Saunders J.E., Crum L.A., Dyson M. (1987). Acoustic cavitation generated by an extracorporeal shockwave lithotripter. Ultrasound Med. Biol..

[21] Bekeredjian R. (2007). Impact of microbubbles on shock wave-mediated DNA uptake in cells in vitro. Ultrasound Med. Biol..

[22] Loske, A. M. Medical and Biomedical Applications of Shock Waves. Medical and Biomedical Applications of Shock Waves (2017).

[23] T. B. Benjamin & A. T. Ellis. A discussion on deformation of solids by the impact of liquids, and its relation to rain damage in aircraft and missiles, to blade erosion in steam turbines, and to cavitation erosion - The collapse of cavitation bubbles and the pressures thereby produced against solid boundaries. *Philosophical Transactions of the Royal Society of London. Series A, Mathematical and Physical Sciences* **260**, (1966).

[24] BRENNEN, C. E. *Cavitation and Bubble Dynamics.* (Oxford University Press).

[25] Sankin G.N., Zhong P. (2006). Interaction between shock wave and single inertial bubbles near an elastic boundary. Phys. Rev. E Stat. Nonlin. Soft Matter Phys..

[26] Crum L.A., Nordling D.A. (1972). Velocity of transient cavities in an acoustic stationary wave. J. Acoust. Soc. Am..

[27] Mettin R., Doinikov A.A. (2009). Translational instability of a spherical bubble in a standing ultrasound wave. Appl. Acoust..

[28] Johnsen E., Colonius T. (2009). Numerical simulations of non-spherical bubble collapse. J. Fluid Mech..

[29] Plesset M.S. (1949). The dynamics of cavitation bubbles. J. Appl. Mech. Trans. ASME.

[30] Keller J.B., Miksis M. (1980). Bubble oscillations of large amplitude. J. Acoust. Soc. Am..

[31] Brenner, M. P., Hilgenfeldt, S. & Lohse, D. Single-bubble sonoluminescence. *Reviews of Modern Physics* vol. 74 Preprint at https://doi.org/10.1103/RevModPhys.74.425 (2002).

[32] Abe A., Wang J., Shioda M., Maeno S. (2015). Observation and analysis of interactive phenomena between microbubbles and underwater shock wave. J. vis. (tokyo)..

[33] Richtmyer R.D. (1960). Taylor instability in shock acceleration of compressible fluids. Commun. Pure Appl. Math..

[34] Kull, H. J. Theory of the Rayleigh-Taylor instability. *Physics Reports* vol. 206 Preprint at https://doi.org/10.1016/0370-1573(91)90153-D (1991).

[35] Brouillette M. (2002). The Richtmyer-Meshkov instability. Annu. Rev. Fluid Mech..

[36] Zhang Q., Sohn S.I. (1996). An analytical nonlinear theory of Richtmyer-Meshkov instability. Phys. Lett. Sec. a: General, Atomic Solid State Phys..

[37] Zhang Q., Guo W. (2015). Universality of finger growth in two-dimensional Rayleigh-Taylor and Richtmyer-Meshkov instabilities with all density ratios. J. Fluid Mech..

[38] Vogel A., Lauterborn W. (1988). Acoustic transient generation by laser-produced cavitation bubbles near solid boundaries. J. Acoust. Soc. Am..

[39] Tomita Y., Shima A. (1986). Mechanisms of impulsive pressure generation and damage pit formation by bubble collapse. J. Fluid Mech..

[40] Philipp A., Delius M., Scheffczyk C., Vogel A., Lauterborn W. (1993). Interaction of lithotripter-generated shock waves with air bubbles. J. Acoust. Soc. Am..

[41] Obreschkow, D. , K. P. , D. N. , de B. A. , N. C. & F. M. Cavitation bubble collapse inside liquid spheres: a model of drop impact cavitation. *Phys. Rev. Lett.* **107**, 204501 (2011).

[42] Gac, S. Le, Zwaan, E., Berg, A. Van Den & Ohl, C. D. Sonoporation of suspension cells with a single cavitation bubble in a microfluidic confinement. *Lab Chip* **7**, (2007). 10.1039/b712897p 18030385

[43] Ohl C.D. (2006). Sonoporation from jetting cavitation bubbles. Biophys. J ..

[44] Zudin Y.B. (1992). Analog of the rayleigh equation for the problem of bubble dynamics in a tube. J. Eng. Phys. Thermophys..

[45] Og̃uz H.N., Prosperetti A. (1998). The natural frequency of oscillation of gas bubbles in tubes. J. Acoust. Soc. Am..

[46] Klotz A.H.K. (2010). Simulations of the Devin and Zudin modified Rayleigh-Plesset equations to model bubble dynamics in a tube. Electron. J. “technical Acoustics”.

[47] Yuan H., Og̃uz H.N., Prosperetti A. (1999). Growth and collapse of a vapor bubble in a small tube. Int. J. Heat Mass Transf..

[48] Wang S.P., Wang Q., Zhang A.M., Stride E. (2019). Experimental observations of the behaviour of a bubble inside a circular rigid tube. Int. J. Multiph. Flow.

[49] Wang N., Xu H., Wang T., Che Z. (2024). Wall confinement effects on the dynamics of cavitation bubbles in thin tubes. Phys. Fluids.

[50] Li J. (2024). Collapsing behavior of spark-induced cavitation bubble in rigid tube. Ultrason. Sonochem..

[51] Gebensleben D., Reuter F., Ohl C.D. (2025). Single cavitation bubble dynamics in a confined planar flow. Int. J. Multiph. Flow.

[52] Wang Z. (2024). Experimental investigations on the cavitation bubble dynamics near the boundary of a narrow gap. Symmetry (basel)..

[53] Rezaee S., Kadivar E., el Moctar O. (2025). Molecular dynamics-based approach for laser-induced cavitation bubbles: bridging experimental and hybrid analytical–computational approaches. Langmuir.

[54] Sharma Y., Ohl C.D., Rosselló J.M. (2024). Nanobubble nucleation by pulsed laser illumination of colloidal gold nanoparticles. Sci. Rep..

[55] Orthaber U., Petkovšek R. (2026). Thermally induced nanobubble filaments and cylindrical shock wave formation in colloidal suspension. Exp. Therm Fluid Sci..

[56] Feng M. (2022). How sodium chloride extends lifetime of bulk nanobubbles in water. Soft Matter.

[57] Vogel A., Lauterborn W., Timm R. (2006). Optical and acoustic investigations of the dynamics of laser-produced cavitation bubbles near a solid boundary. J. Fluid Mech..

[58] Brujan E.A., Keen G.S., Vogel A., Blake J.R. (2002). The final stage of the collapse of a cavitation bubble close to a rigid boundary. Phys. Fluids.

[59] Noack J., Vogel A. (1999). Laser-induced plasma formation in water at nanosecond to femtosecond time scales: calculation of thresholds, absorption coefficients, and energy density. IEEE J. Quantum Electron..

[60] Ohl C.D., Lindau O., Lauterborn W. (1998). Luminescence from spherically and aspherically collapsing laser induced bubbles. Phys. Rev. Lett..

[61] Pierce, A. D. Acoustics: An Introduction to Its Physical Principles and Applications, Third Edition. Acoustics: An Introduction to Its Physical Principles and Applications, Third Edition (2019). doi:10.1007/978-3-030-11214-1.

[62] Kinsler L.E., Frey A.R., Bennett G.S. (1951). Fundamentals of acoustics. Am. J. Phys.

[63] The COMSOL Multiphysics Reference Manual. *COMSOL Multiphysics* 1–1742 (2016).

[64] Staudenraus J., Eisenmenger W. (1993). Fibre-optic probe hydrophone for ultrasonic and shock-wave measurements in water. Ultrasonics.

[65] Koch M. (2016). Numerical modeling of laser generated cavitation bubbles with the finite volume and volume of fluid method, using OpenFOAM. Comput. Fluids.

[66] Lechner C., Lauterborn W., Koch M., Mettin R. (2019). Fast, thin jets from bubbles expanding and collapsing in extreme vicinity to a solid boundary: a numerical study. Phys. Rev. Fluids.

[67] Tagawa Y., Yamamoto S., Hayasaka K., Kameda M. (2016). On pressure impulse of a laser-induced underwater shock wave. J. Fluid Mech..

[68] Han D. (2021). Nanosecond resolution photography system for laser-induced cavitation based on PIV dual-head laser and industrial camera. Ultrason. Sonochem..

[69] Humphrey W., Dalke A., Schulten K.V.M.D. (1996). Visual molecular dynamics. J. Mol. Graph..

[70] Plimpton S. (1995). Fast parallel algorithms for short-range molecular dynamics. J. Comput. Phys..

[71] Martinez L., Andrade R., Birgin E.G., Martínez J.M. (2009). PACKMOL: a package for building initial configurations for molecular dynamics simulations. J. Comput. Chem..

[72] Zavadlav J., Arampatzis G., Koumoutsakos P. (2019). Bayesian selection for coarse-grained models of liquid water. Sci. Rep..

[73] Stuart S.J., Tutein A.B., Harrison J.A. (2000). A reactive potential for hydrocarbons with intermolecular interactions. J. Chem. Phys..

[74] Zhakhovskii V.V., Inogamov N.A., Petrov Y.V., Ashitkov S.I., Nishihara K. (2009). Molecular dynamics simulation of femtosecond ablation and spallation with different interatomic potentials. Appl. Surf. Sci..

[75] Ghoohestani M., Rezaee S., Kadivar E., el Moctar O. (2023). Thermodynamic effects on nanobubble’s collapse-induced erosion using molecular dynamic simulation. Phys. Fluids.

[76] Li C., Chou T.W. (2003). Elastic moduli of multi-walled carbon nanotubes and the effect of van der Waals forces. Compos. Sci. Technol..

[77] Hasan M.N., Shavik S., Rabbi K., Mukut K., Morshed A. (2017). Phase change characteristics of ultra-thin liquid argon film over different flat substrates at high wall superheat for hydrophilic/hydrophobic wetting condition: a non-equilibrium molecular dynamics study. J. Chem. Eng..

[78] Allen M.P., Tildesley D.J. (2017).

[79] Stukowski A. (2010). Visualization and analysis of atomistic simulation data with OVITO-the open visualization tool. Model. Simul. Mat. Sci. Eng..

[80] Alavi S. (2020).

